# Supplementary material for: Mechanical response microRNA-145a-5p alleviates osteoarthritis by inhibiting inflammation and promoting chondrogenesis
Source: PeerJ. 2025 Aug 19;13:e19905. doi: 10.7717/peerj.19905 (PMC12372789; doi:10.7717/peerj.19905)
Supplement: Supplemental Information 3 [file peerj-13-19905-s003.docx]

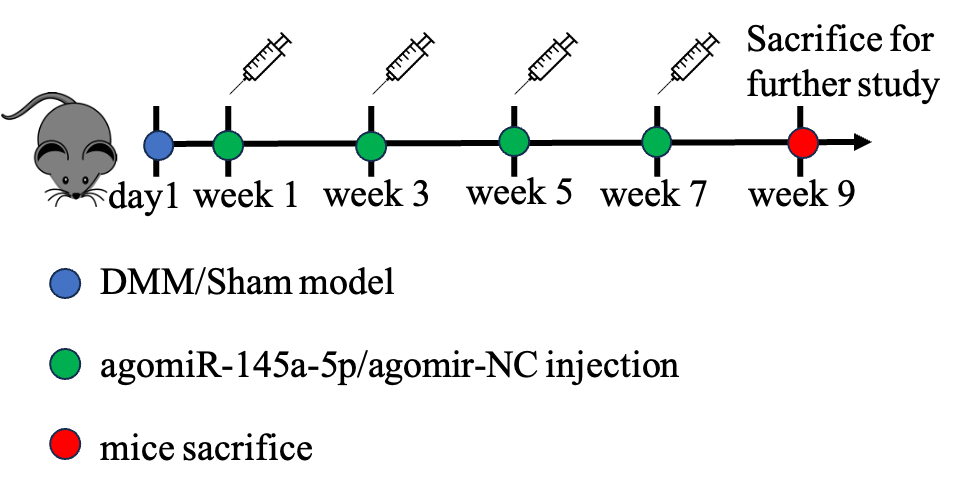


**Supplementary Figure 1: The flowchart illustrates the experimental timeline for DMM surgery.** Agomir-NC (10 µg), or agomir-145a-5p (10 µg) was administered via intra-articular injection at 1-, 3-, 5-, and 7-weeks post-operation. All mice were euthanized nine weeks after the DMM procedure.


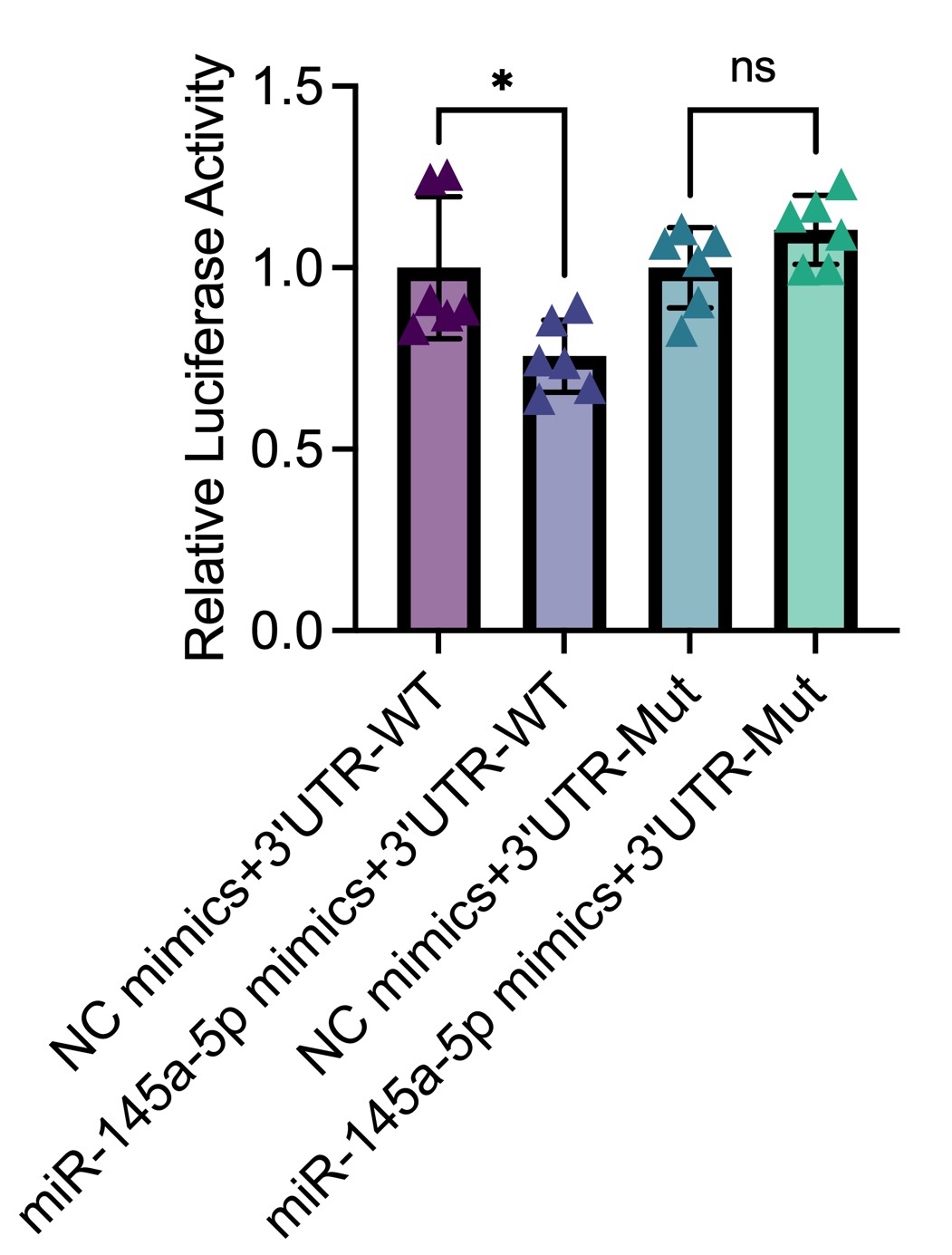


**Supplementary Figure 2: MiR-145a-5p directly modulates IL-6.** Dual-Luciferase reporter assay show repression of luciferase activity by co-transfection of miR-145a-5p mimic with the IL-6 3’UTR-WT reporter, but not with NC mimics or the 3’UTR mutant reporter, confirm that miR-145a-5p directly binds to the 3’UTR of IL-6. (*p < 0.05, **p < 0.01, ***p < 0.005 and ****p < 0.001.)


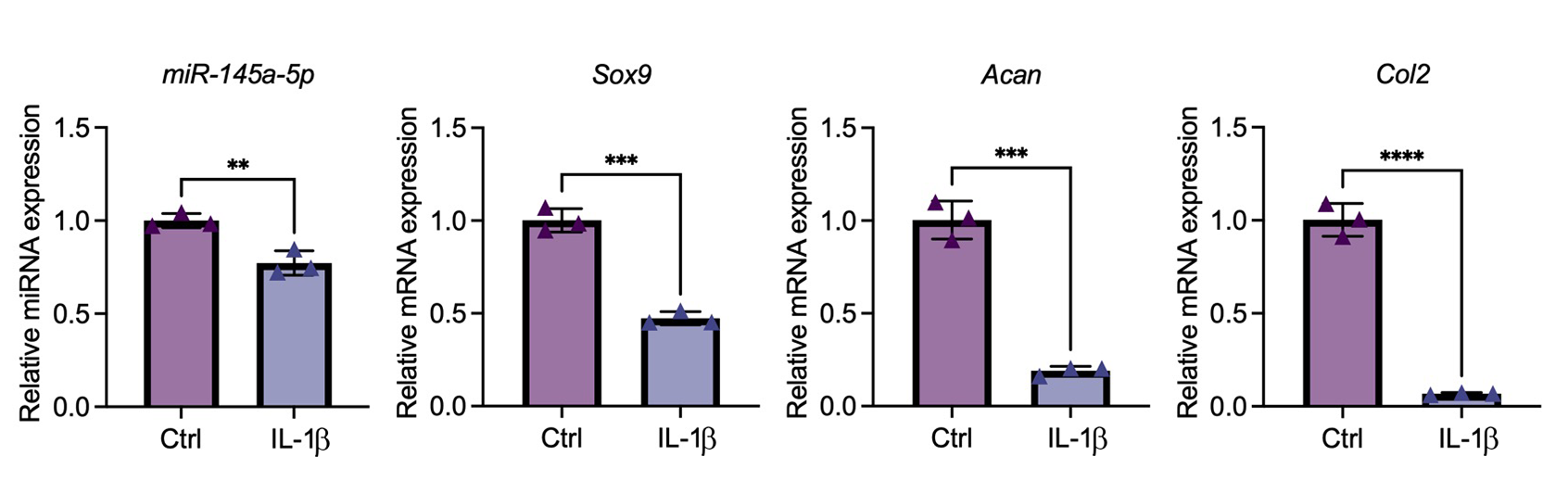


**Supplementary Figure 3: IL-1β suppress the expression level of miR-145a-5p in primary human articular chondrocytes (HACs).** HACs were treated with IL-1β (10ng/ml) and qPCR results show that IL-1β suppress the expression level of *miR-145a-5p, Sox9, Acan,* and *Col2*. (*p < 0.05, **p < 0.01, ***p < 0.005 and ****p < 0.001.)
