# Supplementary figures and images for: Mechanical response microRNA-145a-5p alleviates osteoarthritis by inhibiting inflammation and promoting chondrogenesis
Source: PeerJ. 2025 Aug 19;13:e19905. doi: 10.7717/peerj.19905 (PMC12372789; doi:10.7717/peerj.19905)

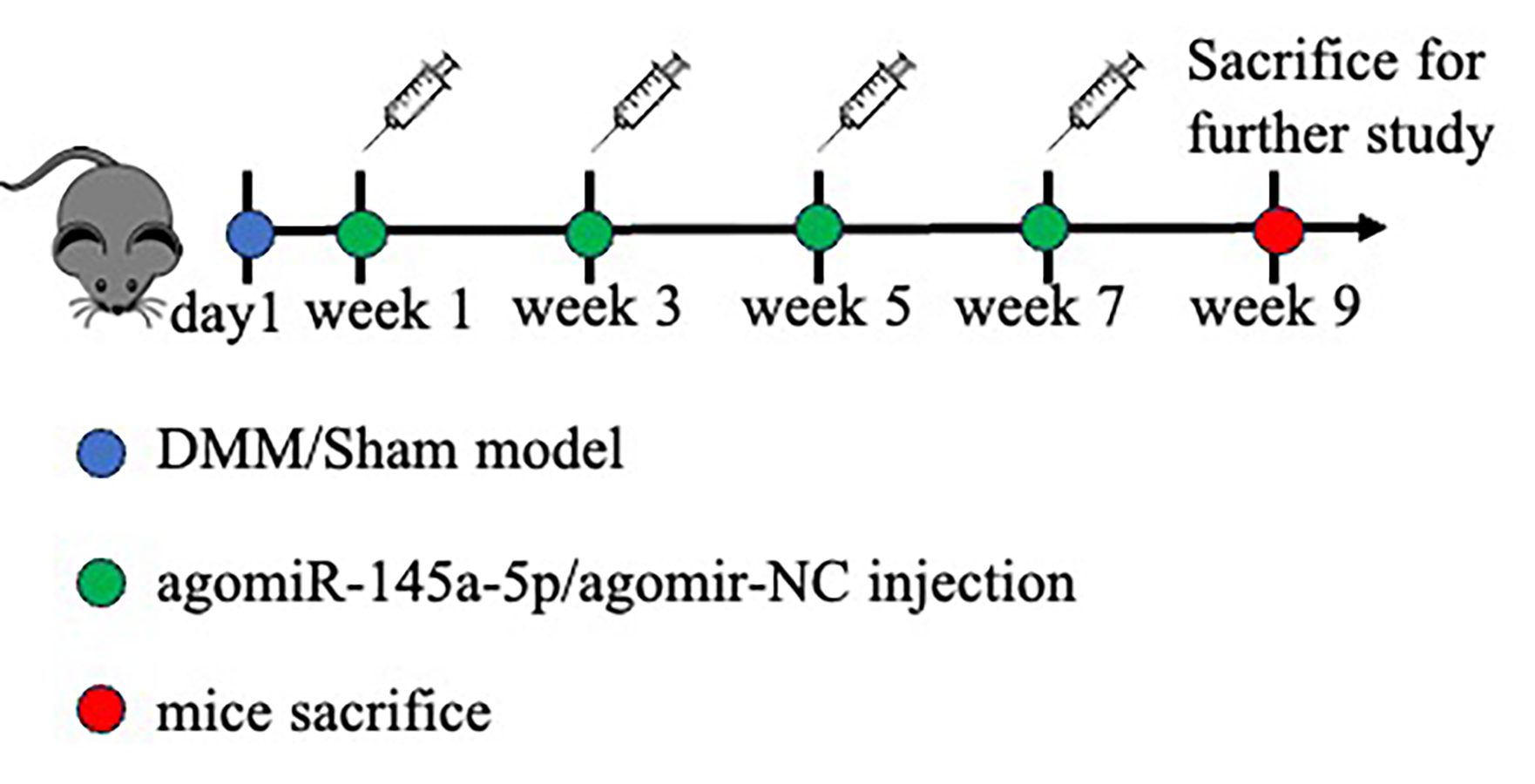

Supplement: Supplemental Information 6 — Agomir-NC (10 µg), or agomir-145a-5p (10 µg) was administered via intra-articular injection at 1-, 3-, 5-, and 7-weeks post-operation. All mice were euthanized nine weeks after the DMM procedure. [file peerj-13-19905-s006.png]

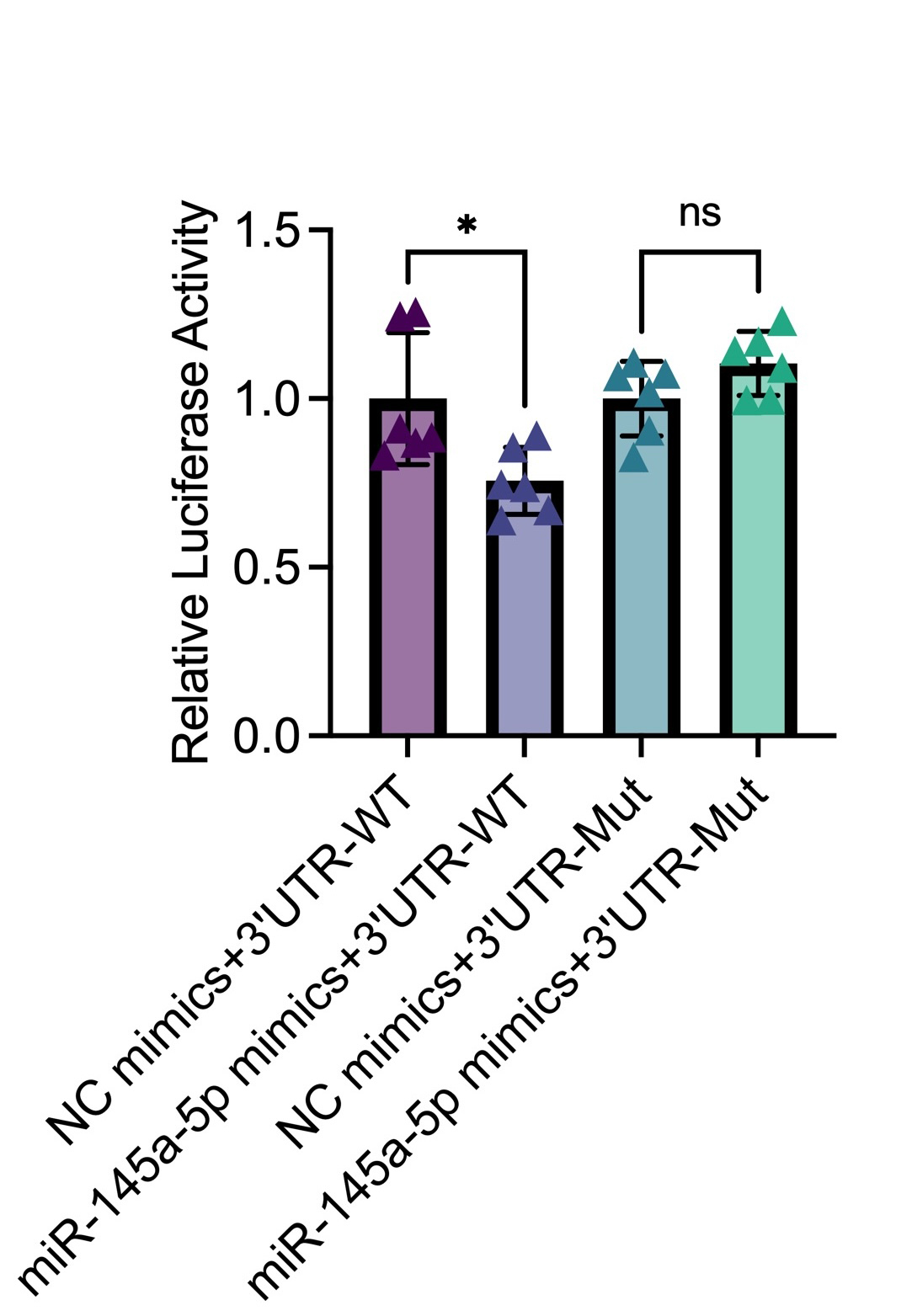

Supplement: Supplemental Information 7 — Dual-Luciferase reporter assay show repression of luciferase activity by co-transfection of miR-145a-5p mimic with the IL-6 3’UTR-WT reporter, but not with NC mimics or the 3’UTR mutant reporter, confirm that miR-145a-5p directly binds to the 3’UTR of IL-6. [file peerj-13-19905-s007.png]

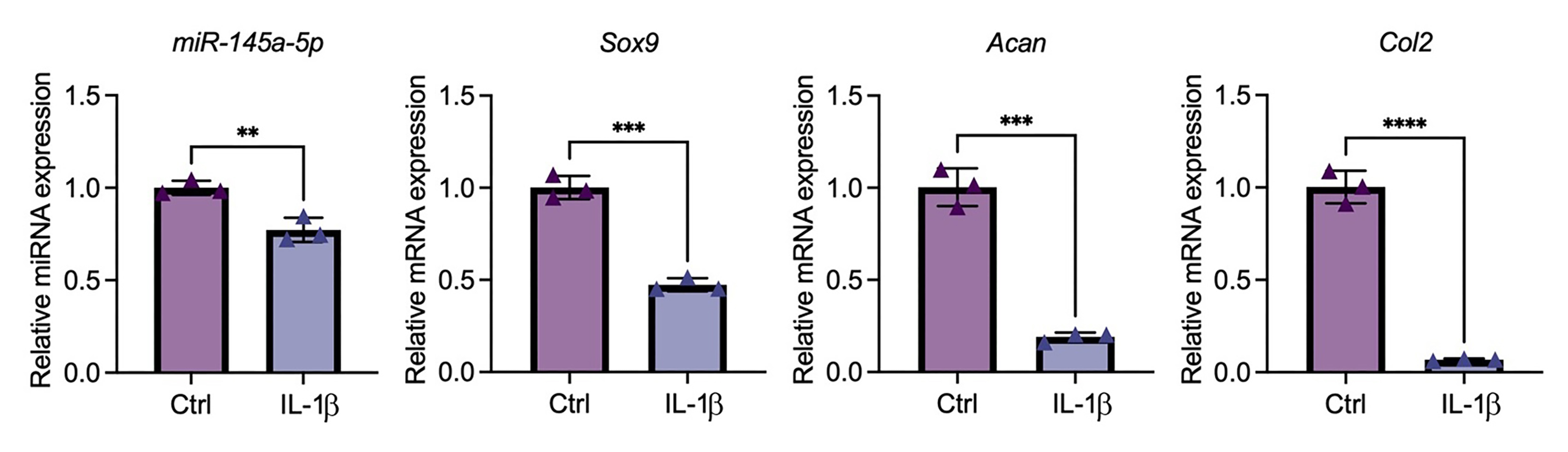

Supplement: Supplemental Information 8 — HACs were treated with IL-1 b (10ng/ml) and qPCR results show that IL-1 b suppress the expression level of miR-145a-5p, Sox9, Acan, and Col2. [file peerj-13-19905-s008.png]
